# The serum levels of specific autoantibodies in systemic sclerosis predict a more severe skin involvement

**DOI:** 10.1177/23971983251357991

**Published:** 2025-07-28

**Authors:** Hannah Dengler, Maya Vonow-Eisenring, Mike Oliver Becker, Rucsandra Dobrota, Carina Mihai, Sinziana Muraru, Anna-Maria Hoffmann-Vold, Oliver Distler, Cosimo Bruni, Muriel Elhai

**Affiliations:** 1 Department of Rheumatology, University Hospital Zurich, University of Zurich, Zurich, Switzerland; 2 Department of Immunology, University Hospital Zurich, University of Zurich, Zurich, Switzerland; 3 Department of Rheumatology, Oslo University Hospital, Oslo, Norway

**Keywords:** Systemic sclerosis, stratification, autoantibodies, precision medicine

## Abstract

**Background:**

Systemic sclerosis is a severe autoimmune disease characterized by fibrosis of the skin and internal organs. Systemic sclerosis is associated with the presence of three specific autoantibodies: anti-topoisomerase I, anti-centromere, and anti-RNA polymerase III autoantibodies, which have also been identified as prognostic factors. However, it remains unknown whether the prognosis also varies based on their serum levels.

**Objectives:**

We aimed to assess the value of serum levels of systemic sclerosis-specific autoantibodies as biomarkers of disease severity and progression in systemic sclerosis.

**Design:**

We conducted a post hoc longitudinal analysis of data of systemic sclerosis patients included in the Zurich EUSTAR cohort, who were positive for at least one of the three systemic sclerosis-specific autoantibodies.

**Methods:**

The association between the levels of systemic sclerosis-specific autoantibodies and disease severity at baseline and during the follow-up was assessed by univariable and multivariable logistic and linear regressions.

**Results:**

The serum levels of anti-topoisomerase I autoantibodies [β = 0.032 (95% confidence interval = 0.014 to 0.049), p < 0.001], anti-centromere [β = 0.002 (95% confidence interval = 0.001 to 0.003), p < 0.001], and anti-RNA polymerase III autoantibodies [β = 0.143 (95% confidence interval = 0.066 to 0.220), p < 0.001] were associated with the modified Rodnan Skin Score in univariable analysis at baseline. For anti-centromere [β = 0.002 (95% confidence interval = 0.001 to 0.003), p < 0.001] and anti-RNA polymerase III autoantibodies [β = 0.135 (95% confidence interval = 0.053 to 0.217), p = 0.002], this association also remained significant in multivariable analysis. In the longitudinal analysis, the levels of the three systemic sclerosis-specific autoantibodies did not predict changes in mRSS over 1 year.

**Conclusion:**

Increased serum levels of all three autoantibodies predicted a more severe skin fibrosis. The results underscore the relevance of measuring the levels of systemic sclerosis-specific autoantibodies to enhance risk stratification in systemic sclerosis, with particular focus on skin involvement.

## Introduction

Systemic sclerosis (SSc) is an autoimmune disease associated with high morbidity and mortality.^1,2^ SSc is characterized by fibrosis affecting the skin and internal organs, along with vasculopathy and immune system dysregulation.^3,4^ SSc is highly heterogeneous, making it difficult to accurately predict the prognosis of individual patients. It is therefore urgent to improve risk stratification, aiming for more personalized management. Antinuclear antibodies are detected in more than 90% of SSc patients.^5–7^ Among them, three predominant and disease-specific autoantibodies are observed: anti-centromere antibodies (ACAs), anti-topoisomerase I (anti-Scl-70) antibodies, and anti-RNA polymerase III (anti-RNA pol III) antibodies.^8^

These antibodies are not only valuable for the diagnosis,^8^ but their presence also serves as strong predictive factors for the further development of a definite SSc in patients with early disease.^5^ Moreover, they are associated with specific subsets of the disease, clinical symptoms, and organ involvement.^9^ ACAs are associated with the limited cutaneous form of the disease (lcSSc), whereas anti-RNA pol III and anti-Scl-70 antibodies are associated with the diffuse cutaneous form (dcSSc).^10,11^ Regarding other organ involvement, anti-Scl-70 are associated with interstitial lung disease (ILD) and digital ulcers.^12^ Anti-RNA pol III are connected with scleroderma renal crisis^13^ and higher risk for malignancy,^14^ whereas patients positive for ACA carry a higher risk of pulmonary arterial hypertension.^15^

In other autoimmune diseases such as rheumatoid arthritis^16^ or antiphospholipid syndrome,^17^ it is well known that the level of autoantibodies has a prognostic value. In SSc, the existence of a pathogenic role of SSc-specific autoantibodies and the prognostic value of their titers remain unknown.^18^ Two studies with limited patient numbers have previously shown a correlation of anti-Scl70 levels with the extent of skin fibrosis in univariable analysis.^19,20^ This association needs, however, to be confirmed in independent larger cohorts and after adjustment for confounders. Moreover, data on the levels of other SSc-specific autoantibodies are still lacking.

Therefore, we aimed to investigate whether the serum levels of the three specific autoantibodies in SSc associate with the severity of organ involvement in a large, well-characterized cohort.

## Materials and methods

### Study design

A post hoc analysis on prospectively collected patient data from the SSc cohort at the University Hospital of Zurich was conducted. All registered patients granted their informed consent to be included in the cohort. The Cantonal Ethic Committee of Zurich (BASEC nos 2016-01515 and 2018-02165) approved the study. This study complied with the Declaration of Helsinki.

### Patient population and characteristics

At the Department of Rheumatology of the University Hospital of Zurich, all consenting patients with a diagnosis of SSc are prospectively included in the local European Scleroderma Trials and Research Group (EUSTAR) database. SSc patients are followed up (at least) once a year and data are recorded.^21^ We queried the Zurich database at the end of April 2023, collecting information on patients ⩾18 years old, enrolled since 2010 and who fulfilled the 2013 classification criteria for SSc by the American College of Rheumatology/European League Against Rheumatism.^8^ The first visit to the center with inclusion in the local EUSTAR cohort was set as the baseline visit. Inclusion criterion was the positivity for one of the SSc-specific autoantibodies, that is, anti-Scl-70, ACA, or anti-RNA pol III autoantibodies. In addition to the collected antibody levels, the following characteristics were recorded: age, sex, disease duration (defined from onset of the first non-Raynaud sign or symptom), presence of Raynaud’s phenomenon, cutaneous subset of SSc,^22^ the extent of skin fibrosis assessed using the modified Rodnan Skin Score (mRSS),^23^ smoking status, presence of digital ulcers, telangiectasia, esophageal (reflux/dysphagia), stomach (early satiety, vomiting), or intestinal (diarrhea, bloating, constipation) symptoms evaluated by medical history, scleroderma renal crisis, joint synovitis, muscle weakness, calcinosis cutis, and scleroderma pattern on capillaroscopy. Lung involvement was assessed by dyspnea stage according to the New York Heart Association (NYHA) functional classification, by the presence of ILD on high-resolution computed tomography (HRCT) and by its extent (⩾20% or <20% on visual quantification). The parameters used to assess lung function included the % predicted of the total lung capacity (TLC), the % predicted of the forced vital capacity (FVC), the % predicted of the diffusion capacity of the lungs for carbon monoxide (DLCO), and the results of the 6-min walking test (including the distance in meters, the oxygen saturation (SpO_2_) at rest, and the worst value of SpO_2_ during exercise). Further parameters included the levels of C-reactive protein (CRP, mg/L), the Health Assessment Questionnaire (HAQ) score, the presence of pulmonary hypertension determined by echocardiography (defined as an elevation of the systolic pulmonary pressure ⩾45 mm Hg), and heart dysfunction defined as left ventricular ejection fraction <50%.

### Levels of SSc-specific autoantibodies

Until 2016, the levels of anti-Scl-70 autoantibodies were manually measured using the qualitative Sclero-Poly-Synthetase Profil 8 Ag IgG Dot (Alphadia). Starting in Mai 2016, these levels were measured quantitatively using the EUROLINE Systemsklerose (Nukleoli)-Profil (IgG) Kit (Euroimmune) automated on the EUROBlotOne System (Euroimmune). The levels of anti-RNA pol III autoantibodies were quantified by enzyme-linked immunosorbent assay (ELISA) with the QUANTA Lite RNA Pol III Assay (INOVA Diagnostics) on the DSX System (Dynex). Anti-RNA pol III antibodies were also available on the EUROLINE Systemsklerose (Nukleoli)-Profil (IgG) Kit (Euroimmune) as RP11 and RP155 subunits. The levels of ACA were measured using the ELIA CENP (IgG) Kit (Phadia AB) which detects antibodies specific to centromere proteins. Antibodies to CENP-B were measured with the UniCap250 system (Thermo Fischer Scientific). Detection of antibodies to CENP-A and CENP-B was available through the EUROLINE Systemic Sclerosis (Nucleoli) Profile. When results for both anti-CENP-A and anti-CENP-B antibodies were available, the higher antibody level was used for the analysis.

### Statistical analysis

The data collected were described using the absolute (n) and the relative frequency (%) for categorical variables. Mean and standard deviation (SD) or median (interquartile range—IQR) were used for continuous variables, according to their distribution. Comparison of characteristics of patients according to their autoantibody status was performed by χ^2^ test and Student’s *t*-test or the Wilcoxon test, according to the distribution of the variable.

We assessed the association between the levels of SSc-specific autoantibodies and disease severity at baseline as reflected by the mRSS, the presence of digital ulcers, the presence and severity of lung fibrosis assessed by FVC using univariable logistic regression analysis, or linear regression analysis. For the longitudinal analysis, we assessed whether the serum levels of SSc-specific autoantibodies at baseline could predict the change in mRSS, DLCO, and FVC over 12 ± 3 months. Odds ratio (OR) or beta regression coefficient (β) and their 95% confidence intervals (CI) were computed to quantify the association. In case of significance, a multivariable regression model adjusted on age, sex, and disease duration was implemented.

All tests were two-sided at a 0.05 significance level. Statistical analyses were carried out using IBM SPSS V29.0.0.0.

## Results

Among 780 patients in the data set at the time of censoring, 563 patients fulfilled the inclusion criteria: 17.4% were males, the mean age was 54.7 ± 14.8 years, and the mean disease duration was 6.2 ± 8.8 years. Overall, 109/532 were positive for anti-Scl-70 autoantibodies, 259/538 ACA, and 47/460 anti-RNA pol III autoantibodies. Further details are provided in Supplemental Table 1.

As expected,^9^ patients with anti-Scl-70 antibodies were more often male, more frequently exhibited dcSSc, had higher prevalence of digital ulcers, ILD, and increased CRP levels. Further comparisons are presented in Supplemental Table 2. Patients with ACA had a milder phenotype with higher female predominance, more limited cutaneous SSc, and lower prevalence of digital ulcers and ILD. Further comparisons are presented in Supplemental Table 3. Patients with anti-RNA pol III antibodies were more often male, with dcSSc and higher frequencies of renal crisis (Supplemental Table 4).

Among these patients, levels of autoantibodies were available in 45 patients with anti-Scl-70 autoantibodies (41% of the anti-Scl-70 group), 244 with ACA (94% of the ACA group), and 45 with anti-RNA pol III antibodies (96% of the anti-RNA pol III group). Further characteristics are presented in Table 1.

**Table 1.** Characteristics of the patients with available levels of SSc autoantibodies at baseline.

Parameter	Patients with anti-Scl-70 antibodies (n = 45)	Patients with ACA (n = 244)	Patients with anti-RNA pol III autoantibodies (n = 45)
Age (years)	51.4 ± 16.2	51.4 ± 16.2	56.5 ± 12.1
Male sex/female sex	15 (33.3%) /30 (66.7%)	23 (9.4%) / 221 (90.6%)	14 (31.1%) / 31 (68.9%)
Disease duration (years)	3.6 ± 5.0	6.4 ± 7.9	5.0 ± 8.3
Raynaud’s present	38 (97.4%)	218 (94.4%)	40 (95.2%)
Diffuse cutaneous SSc	15 (38.5%)	8 (4%)	23 (54.8%)
Cigarette smoking ever	12 (30.8%)	75 (46%)	18 (66.7%)
Digital ulcers ever	14 (32.6%)	26 (14.9%)	4 (12.5%)
Telangiectasia	22 (51.2%)	77 (43.5%)	17 (53.1%)
Esophageal symptoms (dysphagia, reflux)	21 (46.7%)	133 (54.7%)	27 (60%)
Stomach symptoms (early satiety, vomiting)	7 (16.3%)	45 (20.4%)	13 (30.2%)
Intestinal symptoms (diarrhea, bloating, constipation)	9 (20.5%)	59 (26.2%)	9 (20.5%)
mRSS	7.7 ± 10.7	2.00 ± 3.8	9.1 ± 10.3
Renal crisis	0 (0%)	0 (0%)	6 (13.3%)
Dyspnea NYHA stage, II/III	5 (11.1%) / 0 (0%)	30 (12.3%) / 7 (2.9%)	14 (31.1%) / 2 (4.4%)
ILD on HRCT	30 (66.7%)	32 (15.5%)	16 (37.2%)
ILD involvement <20%/>20%	18 (40%)/7 (15.6%)	27 (11.1%) / 3 (1.2%)	6 (13.3%) / 3 (6.7%)
TLC % predicted	85.3 ± 20.6	105.8 ± 16.3	98.7 ± 18.5
FVC % predicted	84.9 ± 21.4	101.5 ± 17.2	93.7 ± 15.4
DLCO % predicted	68.3 ± 28.3	79.7 ± 21.0	69.6 ± 19.4
Six-min walking test (m)	547.3 ± 147.0	529.7 ± 120	512.7 ± 140.9
O_2_ saturation at rest	97.0 ± 5.6	97.3 ± 1.9	96.8 ± 3.0
Worst O_2_ saturation at exercise	95.2 ± 6.1	95.9 ± 4.5	94.9 ± 5.3
LVEF < 50%	0 (0%)	7 (1.4%)	2 (4.8%)
PAP sys (mm Hg)	31.5 ± 17.7	27.6 ± 11.4	26.5 ± 8.5
Pulmonary arterial hypertension^a^	2 (4.4%)	20 (8.2%)	6 (13.3%)
Joint synovitis	6 (13.3%)	43 (17.7%)	13 (28.9%)
Muscle weakness	2 (4.7%)	18 (8.6%)	7 (16.3%)
Calcinosis cutis	3 (9.4%)	10 (8.8%)	1 (5.6%)
CRP (mg/L)	1.0 ± 1.4	0.39 ± 1.00	0.6 ± 1.01
HAQ total score	0.43 ± 0.57	0.4 ± 0.5	0.6 ± 0.7
Scleroderma pattern in the capillaroscopy	24 (70.6%)	85 (57.4%)	12 (52.2%)

Data are mean (SD) or n (%) according to the distribution of the variable.

mRSS, modified Rodnan Skin Score; NYHA, New York Heart Association; TLC, total lung capacity; FVC, forced vital capacity; DLCO, carbon monoxide diffusing capacity; ILD, interstitial lung disease; HRCT, high-resolution computed tomography; LVEF, left ventricular ejection fraction; PAP sys, systolic pulmonary arterial pressure.

a Confirmed on right-heart catheterization.

The levels of anti-Scl-70 antibodies ranged between 17 and 920 U/mL (mean: 133.8 ± 158.4), the levels of ACA between 12 and 3400 U/mL (mean: 432.9 ± 606.3), and the levels of anti-RNA pol III antibodies between 18 and 157 U/mL (mean: 61.3 ± 34.9). For 33 patients, the levels of antibodies to CENP-A and B were analyzed separately with moderate correlation (r = 0.63, p < 0.001). Only two ACA patients showed discordant results regarding reactivity to CENP-A and CENP-B.

We then investigated whether the levels of SSc-specific autoantibodies were associated with markers of disease severity. Anti-Scl-70 levels were associated with the presence of digital ulcers [OR = 1.007 (95% CI = 1.001 to 1.013), p = 0.027] and the mRSS [β = 0.032 (95% CI = 0.014 to 0.049), p < 0.001] in univariable analyses. Interestingly, the association with the mRSS remained significant when considering only patients with dcSSc [β = 0.029 (95% CI = 0.006 to 0.051), p = 0.018]. However, this association did not remain significant in multivariable analysis. No association was observed between anti-Scl-70 levels and FVC or presence of ILD (Tables 2–4 and Supplemental Table 5).

**Table 2.** Predictive value of autoantibody levels on mRSS in univariable and multivariable analyses.

	Anti-Scl-70 autoantibodies		ACA		Anti-RNA pol III autoantibodies	
	Regression coefficient (95% CI)	p	Regression coefficient (95% CI)	p	Regression coefficient (95% CI)	p
mRSS	0.032 (0.014 to 0.049)	< 0.001	0.002 (0.001-0.003)	< 0.001	0.143 (0.066-0.220)	< 0.001
mRSS						
Antibody	0.015 (-0.051 to 0.081)	0.643	0.002 (0.001 to 0.003)	< 0.001	0.134 (0.050 to 0.218)	0.003
Age	-0.036 (-0.248 to 0.176)	0.729	0.016 (-0.033 to 0.064)	0.523	0.156 (-0.114 to 0.426)	0.248
Male sex	3.970 (-3.371 to 11.311)	0.273	0.562 (-1.455 to 2.579)	0.582	1.897 (-4.130 to 7.924)	0.526
Disease	0.375 (-0.316 to 1.066)	0.272	0.083 (0.007 to 0.158)	0.032	-0.062 (-0.437 to 0.314)	0.740
Duration						
CRP levels	2.323 (0.342-4.303)	0.024	0.947 (-0.160 to 2.054)	0.093	-0.169 (-4.244 to 3.906)	0.933
Diffuse SSc	4.806 (-1.372-10.984)	0.121	3.274 (0.346 to 6.201)	0.029	8.461 (2.586 to 14.336)	0.006

mRSS, modified Rodnan skin score; CRP levels (mg/L).

**Table 3.** Predictive value of autoantibody levels on mRSS in patients with diffuse cutaneous form in univariable and multivariable analyses.

	Anti-Scl-70 autoantibodies		ACA		Anti-RNA pol III autoantibodies	
	Regression coefficient (95% CI)	p	Regression coefficient (95% CI)	p	Regression coefficient (95% CI)	p
mRSS	0.029 (0.006 to 0.051)	0.018	0.008 (-0.011 to 0.027)	0.365	0.177 (0.080-0.274)	0.001
mRSS						
Antibody	0.130 (-0.063 to 0.323)	0.156			0.165 (0.046 to 0.285)	0.010
Age	0.012 (-0.467 to 0.490)	0.956			0.205 (-0.221 to 0.631)	0.323
Male sex	-8.502 (-30.512 to 13.508)	0.391			2.859 (-7.384 to 13.103)	0.562
Disease	-0.808 (-2.937 to 1.321)	0.399			-0.247 (-1.037 to 0.543)	0.517
Duration						
CRP	2.156 (-2.340 to 6.651)	0.294			-1.519 (-7.156 to 4.118)	0.576

mRSS, modified Rodnan skin score, CRP levels (mg/L).

**Table 4.** Association of levels of autoantibodies and disease features in univariable and multivariable analyses.

	Anti-Scl-70 autoantibodies		ACA		Anti-RNA pol III autoantibodies	
	Odds ratio (95% CI)	p	Odds ratio (95% CI)	p	Odds ratio (95% CI)	p
Digital ulcers	1.007 (1.001 to 1.013)	0.027	0.001 (0.999 to 1.002)	0.356	0.988 (0.954 to 1.023)	0.486
Digital ulcers						
Antibody	0.979 (0.979 to 1.016)	0.744				
Age	1.031 (0.966 to 1.101)	0.357				
Male sex	3.208 (0.415 to 24.817)	0.264				
Disease duration	1.524 (1.033 to 2.247)	0.034				
ILD	1.001 (0.997 to 1.025)	0.135	1.000 (0.999 to 1.001)	0.725	1.002 (0.985 to 1.020)	0.792
Diffuse cutaneous SSc	1.008 (0.996 to 1.020)	0.199	1.000 (0.999 to 1.001)	0.839	1.012 (0.993 to 1.031)	0.223
Pulmonary arterial hypertension			1.000 (1.000 to 1.001)	0.187		

ILD, interstitial lung disease.

Levels of ACA were associated with mRSS in univariable and multivariable analyses after adjusting for age, sex, and disease duration [β = 0.002 (95% CI = 0.001 to 0.003), p < 0.001]. No other association between ACA levels and disease severity was identified (Tables 2–4 and Supplemental Table 5).

The levels of anti-RNA pol III antibodies were significantly associated with mRSS both in univariable and in multivariable analyses [β = 0.135 (95% CI = 0.053 to 0.217), p = 0.002]. Interestingly, the association remained significant in multivariable analysis also when considering only dcSSc cases [β = 0.167 (95% CI 0.051 to 0.274), p = 0.007]. No other association could be identified (Table 2–4 and Supplemental Table 5).

As a next step, we assessed whether the baseline levels of SSc-specific autoantibodies could predict the change in skin and lung fibrosis over 1 year, assessed by delta mRSS, delta FVC, and delta DLCO. The levels of the three SSc-specific autoantibodies did not predict worsening of skin or lung fibrosis (Supplemental Table 6).

## Discussion

In our study, we could show that increased serum levels of all three autoantibodies predicted a more severe skin fibrosis.

Consistently with previous studies,^19,20,24^ the levels of anti-Scl-70 autoantibodies correlated with the extent of skin fibrosis. However, these previous studies were of small sample size and were not adjusted for confounding factors. Moreover, one of these studies used a semi-quantitative analysis with a cut-off of 240 UI/mL.^25^

In our study, we confirmed this association in an independent cohort, even when the analysis was restricted to patients with dcSSc, where monitoring the mRSS is crucial for both clinical prognosis and clinical trials.^25^

However, in our study, the correlation between anti-Scl-70 autoantibodies and mRSS was not significant anymore after adjusting for age, sex, and disease duration. This could be explained by the small sample size of patients with available data on anti-Scl-70 levels. Supporting a role of anti-Scl-70 levels in disease severity, we also observed an association with digital ulcers, which has not been shown previously. Surprisingly, we could not detect an association between levels of anti-Scl-70 autoantibodies and the presence of ILD or the values of FVC. This result is not in line with previous data coming from a small cohort study, in which the levels of anti-Scl-70 have been associated with decreased FVC.^19^ However, data about ILD in this cohort were missing.

Thus, our data suggest that the level of anti-Scl70 autoantibodies could serve as a marker of disease severity, raising the question of their role in the pathogenesis of the disease. Consistently, disappearance of anti-Scl-70 autoantibodies during the disease has been associated with a favorable outcome.^26–28^

Interestingly, we could demonstrate an association between both the levels of ACA and anti-RNA pol III autoantibodies and the extent of skin fibrosis quantified with the mRSS. Data about the pathogenicity of ACA are rare. In a previous study, elevated levels of ACA were shown to predict the development of definite SSc in very early SSc patients.^9^ Our data strengthen the concept that levels of ACA could be a marker of disease severity. This was not confirmed when only focusing on dcSSc cases; however, ACA-positive dcSSc is quite a rare phenotype, represented in a very limited number in our population (n = 8), and therefore did not allow a well-powered analysis.

Whereas data about pathogenicity of anti-RNA pol III have been published in the context of cancer,^29,30^ no data about pathogenicity of anti-RNA pol III in the context of SSc phenotype have been available until now. We could demonstrate an association between anti-RNA pol III level and the extent of skin fibrosis. Interestingly, this association remained robust also after controlling for confounding factors and only considering the most severe form of the disease, that is, the dcSSc. This highlights the value of anti-RNA pol III level as a possible biomarker of the disease.

Risk stratification is needed in such a heterogeneous disease like SSc. Our study suggests that the levels of SSc-specific autoantibodies could be used to better risk-stratify patients. This more accurate stratification could lead to changes in clinical practice in the future, with adapted monitoring and treatment strategies stratified according to autoantibody levels (e.g. more aggressive treatment in patients with elevated antibodies). Moreover, this is an indirect argument advocating for a pathogenic role of autoantibodies,^18^ suggesting that it is important to develop treatments targeting the humoral autoimmune component in SSc.

Our results suggest different impact of the autoantibody levels on skin fibrosis, as highlighted by the different beta coefficients. Although ACA levels went up to 3400 U/mL, the beta coefficient was small, suggesting a moderate impact on skin fibrosis. On the contrary, anti-Scl-70 and anti-RNA pol III showed higher beta coefficients, suggesting a higher impact on skin fibrosis. These results support the hypothesis of a variable pathogenic effect of the three autoantibodies on skin fibrosis.

Some limitations of this study should be taken into account. First, only IgG serotype levels were available. In a previous study, levels of IgG and IgM ACA were significantly higher in patients with definite SSc as compared to patients with very early SSc.^31^ However, in patients with very early SSc, only IgG ACA levels could predict the evolution to a definite SSc,^31^ highlighting that IgG levels might be more relevant as compared to other isotypes to delineate the prognosis. The assessment of IgG levels in the present study reflects current practice. We had a relatively small group of patients with available anti-Scl-70 antibody levels (n = 45) among the anti-Scl-70 antibody positive group (n = 109). This was explained by the recent availability of a quantitative testing. Despite the small size, the results were significant.

For ACA, two different tests were used. In 33 patients, the Euroline Systemsklerose-Profil was used with levels of antibodies to CENP-A and B analyzed separately. We considered the highest level between the two ACA antibodies, which could indeed have induced a bias. As both markers target closely related centromere components, their concurrent detection is considered potentially redundant. To best capture the most clinically relevant level of autoimmune activity, only the higher titer between antibodies to CENP-A and CENP-B was retained, in line with clinical practice. Moreover, we could not assess longitudinal levels of antibodies in our cohort, because it is not done in routine practice in our center. Further studies should aim to determine whether changes in levels of SSc-specific autoantibodies have a prognostic value, in particular in regard to change in the mRSS.

Despite the limitations, our study has strengths: it is the first study to assess the value of all three SSc-specific autoantibodies simultaneously as biomarkers of severity in SSc. The prevalence of antibodies in our cohort was consistent with the literature.^32^ We performed multivariable analyses and also sensitivity analyses focused only on more severe patients (i.e. dcSSc) to increase the relevance of the analysis, which has not been done in previous studies.

In conclusion, this study suggests that the levels of SSc-specific autoantibodies could improve risk stratification in SSc and might be considered biomarkers of skin disease severity. These results need to be confirmed in further prospective studies, including also longitudinal measurements of the autoantibody levels to assess their sensitivity to change.

## Supplemental Material

sj-pdf-1-jso-10.1177_23971983251357991 – Supplemental material for The serum levels of specific autoantibodies in systemic sclerosis predict a more severe skin involvement Supplemental material, sj-pdf-1-jso-10.1177_23971983251357991 for The serum levels of specific autoantibodies in systemic sclerosis predict a more severe skin involvement by Hannah Dengler, Maya Vonow-Eisenring, Mike Oliver Becker, Rucsandra Dobrota, Carina Mihai, Sinziana Muraru, Anna-Maria Hoffmann-Vold, Oliver Distler, Cosimo Bruni and Muriel Elhai in Journal of Scleroderma and Related Disorders

## References

[1] Elhai M Meune C Avouac J , et al. Trends in mortality in patients with systemic sclerosis over 40 years: a systematic review and meta-analysis of cohort studies. Rheumatology 2012; 51(6): 1017–1026. 21900368 10.1093/rheumatology/ker269

[2] Elhai M Meune C Boubaya M , et al. Mapping and predicting mortality from systemic sclerosis. Ann Rheum Dis 2017; 76(11): 1897–1905. 28835464 10.1136/annrheumdis-2017-211448

[3] Varga J Abraham D. Systemic sclerosis: a prototypic multisystem fibrotic disorder. J Clin Invest 2007; 117(3): 557–567. 17332883 10.1172/JCI31139 PMC1804347

[4] Cutolo M Soldano S Smith V. Pathophysiology of systemic sclerosis: current understanding and new insights. Expert Rev Clin Immunol 2019; 15(7): 753–764. 31046487 10.1080/1744666X.2019.1614915

[5] Koenig M Joyal F Fritzler MJ , et al. Autoantibodies and microvascular damage are independent predictive factors for the progression of Raynaud’s phenomenon to systemic sclerosis: a twenty-year prospective study of 586 patients, with validation of proposed criteria for early systemic sclerosis. Arthritis Rheum 2008; 58(12): 3902–3912. 19035499 10.1002/art.24038

[bibr6-23971983251357991] Walker UA Tyndall A Czirják L , et al. Clinical risk assessment of organ manifestations in systemic sclerosis: a report from the EULAR scleroderma trials and research group database. Ann Rheum Dis 2007; 66(6): 754–763. 17234652 10.1136/ard.2006.062901 PMC1954657

[7] Minier T Guiducci S Bellando-Randone S , et al. Preliminary analysis of the very early diagnosis of systemic sclerosis (VEDOSS) EUSTAR multicentre study: evidence for puffy fingers as a pivotal sign for suspicion of systemic sclerosis. Ann Rheum Dis 2014; 73(12): 2087–2093. 23940211 10.1136/annrheumdis-2013-203716

[8] van den Hoogen F Khanna D Fransen J , et al. 2013 classification criteria for systemic sclerosis: an American College of Rheumatology/European League against rheumatism collaborative initiative. Arthritis Rheum 2013; 65(11): 2737–2747. 24122180 10.1002/art.38098 PMC3930146

[9] Elhai M Sritharan N Boubaya M , et al. Stratification in systemic sclerosis according to autoantibody status versus skin involvement: a study of the prospective EUSTAR cohort. Lancet Rheumatol 2022; 4(11): e785–e794. 10.1016/S2665-9913(22)00217-X 38265945

[10] Allanore Y. Limited cutaneous systemic sclerosis: the unfairly neglected subset. J Scleroderma Relat Disord 2016; 1(3): 241–246.

[11] Nihtyanova SI Denton CP. Autoantibodies as predictive tools in systemic sclerosis. Nat Rev Rheumatol 2010; 6(2): 112–116. 20125179 10.1038/nrrheum.2009.238

[12] Santos CS Morales CM Castro CÁ , et al. Clinical phenotype in scleroderma patients based on autoantibodies. Rheumatol Adv Pract 2023; 7(suppl 1): i26–i33. 10.1093/rap/rkad010 PMC10036993 36968636

[13] Kuwana M Okano Y Pandey JP , et al. Enzyme-linked immunosorbent assay for detection of anti-RNA polymerase III antibody: analytical accuracy and clinical associations in systemic sclerosis. Arthritis Rheum 2005; 52(8): 2425–2432. 16052583 10.1002/art.21232

[14] Shah S Denton CP. Scleroderma autoantibodies in guiding monitoring and treatment decisions. Curr Opin Rheumatol 2022; 34(6): 302–310. 36082759 10.1097/BOR.0000000000000904

[15] Jiang Y Turk MA Pope JE. Factors associated with pulmonary arterial hypertension (PAH) in systemic sclerosis (SSc). Autoimmun Rev 2020; 19(9): 102602. 32659476 10.1016/j.autrev.2020.102602

[16] Aletaha D Neogi T Silman AJ , et al. 2010 Rheumatoid arthritis classification criteria: an American College of Rheumatology/European League against rheumatism collaborative initiative. Arthritis Rheum 2010; 62(9): 2569–2581. 20872595 10.1002/art.27584

[17] Barbhaiya M Zuily S Naden R , et al. The 2023 ACR/EULAR antiphospholipid syndrome classification criteria. Arthritis Rheumatol 2023; 75(10): 1687–1702. 37635643 10.1002/art.42624

[18] Chepy A Bourel L Koether V , et al. Can antinuclear antibodies have a pathogenic role in systemic sclerosis? Front Immunol 2022; 13: 930970. 35837382 10.3389/fimmu.2022.930970 PMC9274282

[19] Sato S Hamaguchi Y Hasegawa M , et al. Clinical significance of anti-topoisomerase I antibody levels determined by ELISA in systemic sclerosis. Rheumatology 2001; 40(10): 1135–1140. 11600743 10.1093/rheumatology/40.10.1135

[20] Hu PQ Fertig N Medsger TA Jr , et al. Correlation of serum anti-DNA topoisomerase I antibody levels with disease severity and activity in systemic sclerosis. Arthritis Rheum 2003; 48(5): 1363–1373. 12746909 10.1002/art.10977

[21] Scheidegger M Boubaya M Garaiman A , et al. Characteristics and disease course of untreated patients with interstitial lung disease associated with systemic sclerosis in a real-life two-centre cohort. RMD Open 2024; 10(1): e003658. 10.1136/rmdopen-2023-003658 PMC10806490 38199606

[22] LeRoy EC Medsger TA. Criteria for the classification of early systemic sclerosis. J Rheumatol 2001; 28(7): 1573–1576. 11469464

[23] Khanna D Furst DE Clements PJ , et al. Standardization of the modified Rodnan skin score for use in clinical trials of systemic sclerosis. J Scleroderma Relat Disord 2017; 2(1): 11–18. 28516167 10.5301/jsrd.5000231 PMC5431585

[24] Tebo AE Schmidt RL Frech TM. Presence of antitopoisomerase I antibody alone may not be sufficient for the diagnosis of systemic sclerosis. J Rheumatol 2019; 46(4): 440–442. 30824655 10.3899/jrheum.180503 PMC7141752

[25] Khanna D Lin CJF Furst DE , et al. Tocilizumab in systemic sclerosis: a randomised, double-blind, placebo-controlled, phase 3 trial. Lancet Respir Med 2020; 8(10): 963–974. 32866440 10.1016/S2213-2600(20)30318-0

[26] Kuwana M Kaburaki J Mimori T , et al. Longitudinal analysis of autoantibody response to topoisomerase I in systemic sclerosis. Arthritis Rheum 2000; 43(5): 1074–1084. 10817562 10.1002/1529-0131(200005)43:5<1074::AID-ANR18>3.0.CO;2-E

[bibr27-23971983251357991] Hamaguchi Y Fujimoto M Hasegawa M , et al. Re-emergence of anti-topoisomerase I antibody with exacerbated development of skin sclerosis in a patient with systemic sclerosis. J Am Acad Dermatol 2010; 62(1): 142–144. 19733937 10.1016/j.jaad.2009.01.032

[28] Merkt W Freitag M Claus M , et al. Third-generation CD19.CAR-T cell-containing combination therapy in Scl70+ systemic sclerosis. Ann Rheum Dis 2024; 83(4): 543–546. 38135464 10.1136/ard-2023-225174 PMC10958299

[29] Xu GJ Shah AA Li MZ , et al. Systematic autoantigen analysis identifies a distinct subtype of scleroderma with coincident cancer. Proc Natl Acad Sci U S A 2016; 113(47): E7526–E7534. 10.1073/pnas.1615990113 PMC5127349 27821747

[30] Schreiber RD Old LJ Smyth MJ. Cancer immunoediting: integrating immunity’s roles in cancer suppression and promotion. Science 2011; 331(6024): 1565–1570. 21436444 10.1126/science.1203486

[31] van Leeuwen NM Boonstra M Bakker JA , et al. Anticentromere antibody levels and isotypes and the development of systemic sclerosis. Arthritis Rheumatol 2021; 73(12): 2338–2347. 34042326 10.1002/art.41814 PMC9297867

[32] Mehra S Walker J Patterson K , et al. Autoantibodies in systemic sclerosis. Autoimmun Rev 2013; 12(3): 340–354. 22743034 10.1016/j.autrev.2012.05.011

