# Supplementary material for: The serum levels of specific autoantibodies in systemic sclerosis predict a more severe skin involvement
Source: J Scleroderma Relat Disord. 2025 Jul 28;10(3):342–9. doi: 10.1177/23971983251357991 (PMC12307331; doi:10.1177/23971983251357991)
Supplement: sj-pdf-1-jso-10.1177_23971983251357991 – Supplemental material for The serum levels of specific autoantibodies in systemic sclerosis predict a more severe skin involvement [file sj-pdf-1-jso-10.1177_23971983251357991.pdf]

Supplementary Table 1: Patient characteristics at baseline

Parameter	All patients (n=563)	N available data
Age (years)	54.7 ± 14.8	563
Male sex	98 (17.4%)	563
Disease duration (years)	6.2 ± 8.8	420
Raynaud's present	292 (51.9%)	317
Diffuse cutaneous SSc	104 (18.5%)	485
Cigarette smoking ever	143 (25.4%)	316
Anti-Scl70 positive	109 (19.4%)	532
Anti-centromere positive	259 (46%)	538
Anti-RNA polymerase III positive	47 (8.3%)	460
Digital ulcers ever	83 (14.7%)	401
Telangiectasia	174 (30.9%)	401
Esophageal symptoms (dysphagia, reflux)	283 (50.3%)	558
Stomach symptoms (early satiety, vomiting)	110 (19.5%)	514
Intestinal symptoms (diarrhea, bloating, constipation)	128 (22.7%)	522
mRSS	4.4 ± 7.2	563
Renal crisis	6 (1.1%)	557
Dyspnea NYHA stage, I/II/III/IV	119 (21.1%) / 93 (16.5%) / 21 (3.7%) / 2 (0.4%)	563
Lung fibrosis, involvement <20%/>20%	94 (16.7%) / 32 (5.7%)	563
TLC % predicted	99.4 ± 19.6	490
FVC % predicted	95.8 ± 19.7	507
DLCO % predicted	75.8 ± 21.8	536
ILD on HRCT	147 (28.2%)	498
6 min walking test (m)	527.6 ± 123.5	410
O2 Saturation at rest	97.1 ± 2.9	403
Worst O2 Saturation at exercise	95.1 ± 5.7	396
LVEF<50%	7 (1.2%)	524
PAP sys (mmHg)	27.8 ± 11.2	407
Joint synovitis	107 (19%)	557
Muscle weakness	65 (11.5%)	500
Calcinosis cutis	17 (3%)	240
CRP (mg/L)	0.5 ± 1.1	546
HAQ total score	0.45 ± 0.58	118
Scleroderma pattern in the capillaroscopy	194 (34.5%)	315

Data are mean (SD) or n (%) according to the distribution of the variable. Abbreviations: mRSS, modified Rodnan Skin Score; NYHA, New York Heart Association; TLC, total lung capacity; FVC, forced vital capacity; DLCO, carbon monoxide diffusing capacity; ILD, interstitial lung disease; HRCT, high resolution computed tomography; LVEF, left ventricular ejection fraction; PAP sys, systolic pulmonary arterial pressure

Supplementary Table 2: Comparison of patients with anti-Scl70 autoantibodies and without anti-Scl70 autoantibodies

Parameter	Patients with anti-Scl70 antibodies (n=45)	Patients without anti-Scl70 antibodies (n=473)	P value	N available data
Age (years)	51.4 ± 16.2	54.8 ± 14.7	0.366	518
Male sex	15 (33.3%) /30 (66.7%)	74 (15.6%) / 399 (84.4%)	0.005	518
Disease duration (years)	3.6 ± 5.0	6.2 ± 9.0	0.037	388
Raynaud's present	11 (100%)	259 (91.9%)	0.611	284
Diffuse cutaneous SSc	15 (38.5%)	78 (19.1%)	0.007	447
Cigarette smoking ever	12 (30.8%)	122 (46.4%)	0.084	302
Digital ulcers ever	14 (32.6%)	62 (18.6%)	0.043	376
Telangiectasia	22 (51.2%)	143 (42.9%)	0.330	376
Esophageal symptoms	21 (46.7%)	240 (51.1%)	0.641	515
Stomach symptoms	7 (16.3%)	95 (21.9%)	0.443	476
Intestinal symptoms	9 (20.5%)	108 (24.8%)	0.586	480
mRSS	7.7 ± 10.7	4.1 ± 6.8	<0.001	518
Renal crisis	0 (0%)	5 (1.1%)	1.000	512
Dyspnea NYHA stage (I/II/III/IV)	5 (11.1%) / 5 (11.1%) / 0 (0%) / 0 (0%)	102 (21.6%) / 76 (16.1%) / 20 (4.2%) / 2 (0.4%)	0.108	518
Lung fibrosis, involvement <20%/>20%	18 (40%)/7 (15.6%)	70 (14.8%)/23 (4.9%)	<0.001	518
TLC % predicted	85.3 ± 20.6	100.7 ± 19.1	0.332	453
FVC % predicted	84.9 ± 21.4	96.9 ± 19.1	0.197	468
DLCO % predicted	68.3 ± 28.3	76.1 ± 21.2	0.008	493
ILD on HRCT	30 (66.7%)	130 (30.9%)	<0.001	466
6 min walking test (m)	547.3 ± 147.0	524.9 ± 122.6	0.143	385
O2 Saturation at rest	97.0 ± 5.6	97.2 ± 2.4	0.003	378
Worst O2 Saturation at exercise	95.2 ± 6.1	95.3 ± 5.6	0.415	372
LVEF <50%	0 (0%)	7 (1.6%)	0.635	486
PAP sys (mmHg)	31.5 ± 17.7	27.6 ± 10.7	0.087	375
Joint synovitis	6 (13.3%)	91 (18.5%)	0.334	512
Muscle weakness	2 (4.7%)	60 (14.4%)	0.098	461
Calcinosis cutis	3 (9.4%)	13 (6.7%)	0.707	226
CRP (mg/L)	1.0 ± 1.4	0.5 ± 1.0	<0.001	505
HAQ	0.43 ± 0.57	0.46 ± 0.59	0.656	117
Scleroderma pattern in the capillaroscopy	24 (70.6%)	154 (60.4%)	0.268	289

Data are mean (SD) or n (%) according to the distribution of the variable. Abbreviations: mRSS, modified Rodnan Skin Score; NYHA, New York Heart Association; TLC, total lung capacity; FVC, forced vital capacity; DLCO, carbon monoxide diffusing capacity; ILD, interstitial lung disease; HRCT, high resolution computed tomography; LVEF, left ventricular ejection fraction; PAP sys, systolic pulmonary arterial pressure; p values were obtained using student's t-test or Chi-square test

Supplementary Table 3: Comparison of patients with ACA and without ACA

Variable	Patients with ACA (n=244)	Patients without ACA (n=278)	P value	N available data
Age (years)	51.4 ± 16.2	54.8 ± 14.7	0.481	522
Male sex	23 (9.4%) / 221 (90.6%)	70 (25.2%) / 208 (74.8%)	<0.001	522
Disease duration (years)	6.4 ± 7.9	5.5 ± 9.3	0.94	385
Raynaud's present	118 (90.8%)	146 (93.6%)	0.505	286
Diffuse cutaneous SSc	8 (4%)	87 (35.5%)	<0.001	447
Cigarette smoking ever	75 (46%)	62 (44%)	0.730	304
Digital ulcers ever	26 (14.9%)	54 (26.6%)	0.006	378
Telangiectasia	77 (43.5%)	88 (43.8%)	1.000	378
Esophageal symptoms	133 (54.7%)	129 (46.7%)	0.079	519
Stomach symptoms	45 (20.4%)	555 (21.3%)	0.822	479
Intestinal symptoms	59 (26.2%)	56 (21.5%)	0.24	485
mRSS	2.00 ± 3.8	6.5 ± 8.8	<0.001	522
Renal crisis	0 (0%)	5 (1.8%)	0.063	516
Dyspnea NYHA stage (I/II/III/IV)	51 (20.9%) / 30 (12.3%) / 7 (2.9%) / 0 (0%)	58 (20.9%) / 51 (18.3%) / 12 (4.3%) / 2 (0.7%)	0.134	522
Lung fibrosis, involvement <20%/>20%	27 (11.1%) / 3 (1.2%)	60 (21.6%) / 27 (9.7%)	<0.001	522
TLC % predicted	105.8 ± 16.3	93.3 ± 20.1	<0.001	451
FVC % predicted	101.5 ± 17.2	90.2 ± 19.6	0.09	468
DLCO % predicted	79.7 ± 21.0	72.2 ± 22.3	0.420	496
ILD on HRCT	32 (15.5%)	123 (48%)	<0.001	463
6 min walking test (m)	529.7 ± 120	528 ± 128.6	0.616	380
O2 Saturation at rest	97.3 ± 1.9	97 ± 3.7	0.059	374
Worst O2 Saturation at exercise	95.9 ± 4.5	94.8 ± 1.9	0.002	369
LVEF <50%	7 (1.4%)	4 (1.5%)	1.000	490
PAP sys (mmHg)	27.6 ± 11.4	28.1 ± 11.5	0.816	379
Joint synovitis	43 (17.7%)	55 (20.1%)	0.502	516
Muscle weakness	18 (8.6%)	41 (16.1%)	0.017	464
Calcinosis cutis	10 (8.8%)	7 (6%)	0.459	229
CRP (mg/L)	0.39 ± 1.00	0.6 ± 1.2	<0.001	507
HAQ	0.4 ± 0.5	0.5 ± 0.6	0.113	115
Scleroderma pattern in the capillaroscopy	85 (57.4%)	97 (65.1%)	0.191	297

Data are mean (SD) or n (%) according to the distribution of the variable. Abbreviations: mRSS, modified Rodnan Skin Score; NYHA, New York Heart Association; TLC, total lung capacity; FVC, forced vital capacity; DLCO, carbon monoxide diffusing capacity; ILD, interstitial lung disease; HRCT, high resolution computed tomography; LVEF, left ventricular ejection fraction; PAP sys, systolic pulmonary arterial pressure; p values were obtained using student's t-test or Chi-square test

Supplementary Table 4: Comparison of patients with anti-RNA polymerase III autoantibodies and without anti-RNA polymerase III autoantibodies

Variable	Patients with ARA antibodies (n=45)	Patients without ARA antibodies (n=452)	P value	N available data
Age (years)	56.5 ± 12.1	54.2 ± 15.2	0.064	497
Male sex	14 (31.1%) / 31 (68.9%)	76 (16.8%) / 376 (83.2%)	0.025	497
Disease duration (years)	5.0 ± 8.3	6.2 ± 8.9	0.654	364
Raynaud's present	26 (100%)	216 (92.7%)	0.232	259
Diffuse cutaneous SSc	23 (54.8%)	72 (18.4%)	<0.001	433
Cigarette smoking ever	18 (66.7%)	123 (44.4%)	0.042	304
Digital ulcers ever	4 (12.5%)	75 (21%)	0.359	389
Telangiectasia	17 (53.1%)	148 (41.7%)	0.263	387
Esophageal symptoms	27 (60%)	215 (48.1%)	0.159	492
Stomach symptoms	13 (30.2%)	85 (20.9%)	0.174	450
Intestinal symptoms	9 (20.5%)	104 (25.2%)	0.584	457
mRSS	9.1 ± 10.3	3.5 ± 6.3	<0.001	497
Renal crisis	6 (13.3%)	0 (0%)	<0.001	491
Dyspnea NYHA stage (I/II/III/IV)	6 (13.3%) / 14 (31.1%) / 2 (4.4%) / 0 (0%)	101 (26.5%) / 68 (17.8%) / 11 (2.9%) / 2 (0.5%)	0.097	497
Lung fibrosis, involvement <20%/>20%	6 (13.3%) / 3 (6.7%)	84 (18.6%) / 27 (6%)	0.776	497
TLC % predicted	98.7 ± 18.5	99.5 ± 19.7	0.969	464
FVC % predicted	93.7 ± 15.4	95.9 ± 20.2	0.090	479
DLCO % predicted	69.6 ± 19.4	76.3 ± 22.0	0.292	479
ILD on HRCT	16 (37.2%)	141 (33.9%)	0.736	459
6 min walking test (m)	512.7 ± 140.9	531.1 ± 120.7	0.589	393
O2 Saturation at rest	96.8 ± 3.0	95.3 ± 5.7	0.382	386
Worst O2 Saturation at exercise	94.9 ± 5.3	95.3 ± 5.7	0.886	379
LVEF <50%	2 (4.8%)	5 (1.2%)	0.12	476
PAP sys (mmHg)	26.5 ± 8.5	27.2 ± 10.1	0.69	365
Joint synovitis	13 (28.9%)	81 (18.2%)	0.109	491
Muscle weakness	7 (16.3%)	49 (12.4%)	0.471	438
Calcinosis cutis	1 (5.6%)	14 (6.5%)	1.000	233
CRP (mg/L)	0.6 ± 1.01	0.5 ± 1.06	0.481	482
HAQ	0.6 ± 0.7	0.4 ± 0.6	0.09	113
Scleroderma pattern in the capillaroscopy	12 (52.2%)	167 (61.4%)	0.506	295

Data are mean (SD) or n (%) according to the distribution of the variable. Abbreviations: mRSS, modified Rodnan Skin Score; NYHA, New York Heart Association; TLC, total lung capacity; FVC, forced vital capacity; DLCO, carbon monoxide diffusing capacity; ILD, interstitial lung disease; HRCT, high resolution computed tomography; LVEF, left ventricular ejection fraction; PAP sys, systolic pulmonary arterial pressure; p values were obtained using student's t-test or Chi-square test

Supplementary Table 5: Association of levels of antibodies with FVC in patients with ILD antibodies in univariable analysis

	Anti-Scl70 autoantibodies		ACA		Anti-RNA pol III autoantibodies	
	Regression coefficient (95% CI)	P value	Regression coefficient (95% CI)	P value	Regression coefficient (95% CI)	P value
FVC	0.056 (-0.103 to 0.216)	0.475	0.009 (-0.006 to 0.025)	0.221	0.076 (-0.254 to 0.407)	0.627

Abbreviations: FVC, forced vital capacity; ILD, interstitial lung disease

Supplementary Table 6: Association of levels of antibodies at baseline with progression of skin and lung fibrosis over 1 year in univariable and multivariable analysis

	Anti-Scl70 levels at baseline		ACA levels at baseline		Anti-RNA pol III levels at baseline	
	Regression coefficient (95% CI)	P value	Regression coefficient (95% CI)	P value	Regression coefficient (95% CI)	P value
Delta mRSS	-0.009 (-0.25 to 0.008)	0.3	0.000 (-0.001 to 0)	0.553	-0.006 (-0.074 to 0.062)	0.856
Delta mRSS<br>-antibody<br>-disease duration<br>-diffuse	 -0.055 (-0.119 to 0.010)<br>-0.059 (-0.505 to 0.387)<br>0.996 (-4.054 to 6.045)	 0.093<br>0.782<br>0.679	 -0.001 (-0.002 to 0)<br>0.007 (-0.056 to 0.07)<br>-1.170 (-3.393 to 1.05)	 0.256<br>0.826<br>0.298	 0.004 (-0.075 to 0.084)<br>0.065 (-0.213 to 0.344)<br>-5.724 (-10.498 to -0.949)	 0.907<br>0.632<br>0.021
Delta DLCO	-0.011 (-0.167 to 0.145)	0.884	0.001 (-0.003 to 0.006)	0.587	-0.038 (-0.155 to 0.079)	0.514
Delta DLCO<br>-antibody<br>-ILD<br>-disease duration	 -0.007 (-0.203 to 0.188)<br>5.869 (-11.322 to 23.059)<br>0.417 (-0.561 to 1.396)	 0.938<br>0.478<br>0.378	 0.001 (-0.005 to 0.006)<br>6.384 (-1.815 to 14.58)<br>0.128 (-0.209 to 0.466)	 0.761<br>0.125<br>0.450	 -0.099 (-0.226 to 0.028)<br>-8.722 (-17.424 to -0.019)<br>-0.284 (-0.716 to 0.149)	 0.121<br>0.050<br>0.187
Delta FVC	0.026 (-0.076 to 0.128)	0.604	0.000 (-0.005 to 0.004)	0.850	-0.131 (-0.306 to 0.043)	0.135
Delta FVC<br>-antibody<br>-ILD<br>-disease duration	 0.25 (-0.109 to 0.159)<br>3.503 (-8.278 to 15.284)<br>0.171 (-0.499 to 0.842)	 0.697<br>0.536<br>0.595	 0.00 (-0.006 to 0.006)<br>1.738 (-6.609 to 10.08)<br>0.086 (-0.257 to 0.429)	 0.997<br>0.679<br>0.619	 -0.176 (-0.375 to 0.022)<br>-7.765 (-21.154 to 5.623)<br>-0.338 (-0.995 to 0.319)	 0.079<br>0.241<br>0.297

Abbreviations: mRSS, modified Rodnan; FVC, forced vital capacity; DLCO, carbon monoxide diffusing capacity; ILD, interstitial lung disease
